# Mastoid effusion on temporal bone MRI in patients with Bell’s palsy and Ramsay Hunt syndrome

**DOI:** 10.1038/s41598-021-82984-w

**Published:** 2021-02-04

**Authors:** Jin Woo Choi, Jiyeon. Lee, Dong-Han Lee, Jung Eun Shin, Chang-Hee Kim

**Affiliations:** 1 Department of Radiology, Konkuk University Medical Center, Research Institute of Medical Science, Konkuk University School of Medicine, Seoul, Republic of Korea; 2 Department of Otorhinolaryngology-Head and Neck Surgery, Konkuk University Medical Center, Research Institute of Medical Science, Konkuk University School of Medicine, 120-1 Neungdong-ro, Gwangjin-gu, Seoul, 05030 Republic of Korea

**Keywords:** Diseases, Medical research, Neurology, Signs and symptoms

## Abstract

This study aimed to investigate the incidence of mastoid effusion on temporal bone magnetic resonance imaging (MRI) in patients with Bell’s palsy (BP) and Ramsay Hunt syndrome (RHS), and evaluate the usefulness of mastoid effusion in early differential diagnosis between BP and RHS. The incidence of mastoid effusion on 3.0 T—temporal bone MRI, which was conducted within 10 days after the onset of acute facial nerve palsy, was compared between 131 patients with BP and 33 patients with RHS. Findings of mastoid cavity on temporal bone MRI were classified into three groups as normal mastoid, mastoid effusion, and sclerotic change, and the incidence of ipsilesional mastoid effusion was significantly higher in RHS than BP (*P* < 0.001). Tympanic membrane was normal in 7 of 14 RHS patients with mastoid effusion, and injected without middle ear effusion in 7 patients. This study highlights significantly higher incidence of ipsilesional mastoid effusion in RHS than BP, and suggests that the presence of mastoid effusion may provide additional information for differential diagnosis between RHS and BP.

## Introduction

Bell’s palsy (BP) is the most common type of acute peripheral facial nerve palsy (FNP) with an annual incidence of 20–30 per 100,000 persons^1^, and Ramsay Hunt syndrome (RHS) accounts for approximately 10% of acute FNP with an annual incidence of 5 per 100,000 population^2^. RHS is characterized by otalgia and erythematous vesicular rash in the external auditory meatus or auricle in addition to acute FNP. While vertigo or hearing loss is commonly accompanied with FNP in RHS, symptoms of vestibulocochlear deficit is rare in BP^3^. In comparison to BP, the severity of FNP is greater and recovery rate is worse in RHS^4,5^. On the other hand, the gustatory perception and morphology may be bilaterally affected in both BP and RHS at the acute phase^6^. It has been established that combination therapy with systemic steroids and antiviral agents shows benefit over systemic steroids alone in RHS^7^, whereas the additional benefit from combination therapy over steroids alone has not been confirmed in BP^8^. Several prognostic factors for the recovery of FNP have been reported, and early treatment was suggested as one of the most important prognostic factors in RHS^9,10^. Thus, initiating early treatment after proper diagnosis is crucial to improve the recovery rate in RHL. However, differential diagnosis between RHS and BP at an early stage is sometimes difficult because the sequence, in which symptoms appear, may vary among individual patients with RHS. For example, acute FNP may precede erythematous skin rash in some patients with RHS^11,12^. Moreover, in cases such as zoster sine herpete, in which acute FNP develops without erythematous skin rash, differentiation of RHS from BP based on clinical presentation is difficult.

Temporal bone magnetic resonance imaging (MRI) has been utilized to evaluate disease extent of RHS^13–17^ and compare imaging characteristics between RHS and BP^18–20^. Most of all, temporal bone MRI is helpful for the differential diagnosis between BP and RHS, facilitating early diagnosis of RHS and supporting rapid initiation of combination therapy with systemic steroids and antiviral agents^20^. We accidentally discovered mastoid effusion on the ipsilesional side of FNP in one patient with RHS, and attempted to retrospectively investigate if presence of mastoid effusion in temporal bone MRI may provide additional information for differential diagnosis between RHS and BP. The purpose of the present study was to investigate the incidence of mastoid effusion on temporal bone MRI in patients with BP and RHS, and evaluate the usefulness of mastoid effusion in differential diagnosis between BP and RHS.

## Subjects and methods

Between February 2013 and June 2018, patients diagnosed with BP or RHS were enrolled in this study. A retrospective analysis of the charts and MRI findings of 131 patients with BP (65 men and 66 women; mean age = 45 years; age range = 10–84 years) and 33 patients with RHS (14 men and 19 women; mean age = 53 years; age range = 22–88 years) was performed (Table 1). Patients with acute unilateral FNP, who underwent temporal bone MRI within 10 days after the symptom onset, were included in the present study. Patients with acute FNP with otalgia and vesicular skin eruption were diagnosed with RHS, and those with no other known cause of FNP were diagnosed with BP. Neurological and otolaryngological examinations were performed, and patients with chronic otitis media, otitis media with effusion, recent head or ear trauma, or a prior history of otologic surgery in either ear were excluded from the study. The study was approved by the Institutional Review Board of Konkuk University Medical Center (approval number: 1110083). Informed consent was waived by the same IRB that approved the study. All procedures performed in studies involving human participants were in accordance with the ethical standards of the institutional and/or national research committee and with the 1964 Helsinki declaration and its later amendments or comparable ethical standards.

**Table 1:** Summary of clinical characteristics.

Characteristics	BP (*n* = 131)	RHS (*n* = 33)	
Male: female	65: 66	14 : 19	*P* = 0.460
Mean age (range)	45 years (10 -84 years)	53 years (22–88 years)	*P* = 0.369
Affected side (Right : Left)	63 : 68	15 : 18	*P* = 0.786
From onset of FNP to MRI	2.9 ± 2.7 days	2.7 ± 2.6 days	*P* = 0.821
House-Brackmann grade	3.2 ± 0.8	3.6 ± 0.9	*P* = 0.005
No. of patients with hearing loss	0 (0%)	29 (88%)	*P* < 0.001
No. of patients with vertigo	0 (0%)	24 (73%)	*P* < 0.001

BP, Bell’s palsy; RHS, Ramsay Hunt syndrome; FNP, facial nerve palsy; MRI, magnetic resonance imaging.

Temporal bone MRI was conducted using a 3.0 T MRI (Signa HDx: GE healthcare, Milwaukee, WI) with a phased-array head coil. We used the same temporal bone MRI protocol as our previous studies^14,21^, which included 2-mm thick T1 (TR/TE on 3.0 T MRI; 600/14 ms and 800/2 ms) and T2 (TR/TE; 4000/92 ms and 4000/107 ms) weighted axial images, 3D balanced steady-state gradient echo sequence (FIESTA) for cranial nerves, and post-contrast 3D volumetric T1-weighted images (SPGR). The parameters for acquiring FIESTA data for cranial nerves on 3.0 T MRI were the following: TR, 6.1/7.28 ms; TE, 1.7/2.30 ms; FOV, 180 mm; flip angle, 658/608; matrix, 512 256; acquiring slice thickness, 1 mm; reconstructed slice thickness, 0.5 mm. Postcontrast 3D volumetric T1-weighted images (TR/TE, 8.85/3.95 ms; FOV, 180 mm; flip angle, 128; matrix, 256 256; acquiring slice thickness, 1.2 mm; reconstructed slice thickness, 0.6 mm) were obtained at 1 min after an intravenous bolus injection of a standard dose of gadobutrol (Gadovist; Schering, Berlin, Germany; 0.1 mmol/kg of body weight) through the antecubital vein using a power injector with a rate of 1 mL per second. All injections were followed by a saline flush of up to 20 mL. The scanning encompassed the region from the mastoid to the upper edge of the petrous bone.

Statistical comparison was performed using Pearson’s chi-squared test for categorical variables and Student’s *t*-test or Mann – Whitney *U* test for ordinal or interval variables (IBM SPSS version 23.0, IBM Corp., Armonk, NY), and *P* < 0.05 was considered significant.

### Ethics declaration

The study was approved by the Institutional Review Board (Approval Number: 1110083). All procedures performed in studies involving human participants were in accordance with the ethical standards of the institutional and/or national research committee and with the 1964 Helsinki declaration and its later amendments or comparable ethical standards.

## Results

The clinical characteristics in patients with BP and RHS are summarized in Table 1. Male to Female ratio was 65 : 66 in BP and 14 : 19 in RHS, which was not significantly different (*P* = 0.460, Pearson’s chi-squared test). Mean age was 45 years in BP and 53 years in RHS, which was not significantly different (*P* = 0.369, Student’s *t*-test). Affected side was right in 63 patients with BP (left side in 68 patients) and 15 patients with RHS (left side in 18 patients), which was not significantly different (*P* = 0.786, Pearson’s chi-squared test). The time period from symptom onset to temporal bone MRI was 2.9 ± 2.7 days in BP and 2.7 ± 2.6 days in RHS, which was not significantly different (*P* = 0.821, Student’s *t*-test). The worst House-Brackmann (HB) grade during an acute stage was significantly higher in RHS (3.6 ± 0.9) than BP (3.2 ± 0.8) (*P* = 0.005, Student’s *t*-test). Proportion of patients with hearing loss was significantly higher in RHS (88%) than BP (0%) (*P* < 0.001, Pearson’s chi-squared test). Proportion of patients with vertigo was significantly higher in RHS (73%) than BP (0%) (*P* < 0.001, Pearson’s chi-squared test).

**Figure 1:**
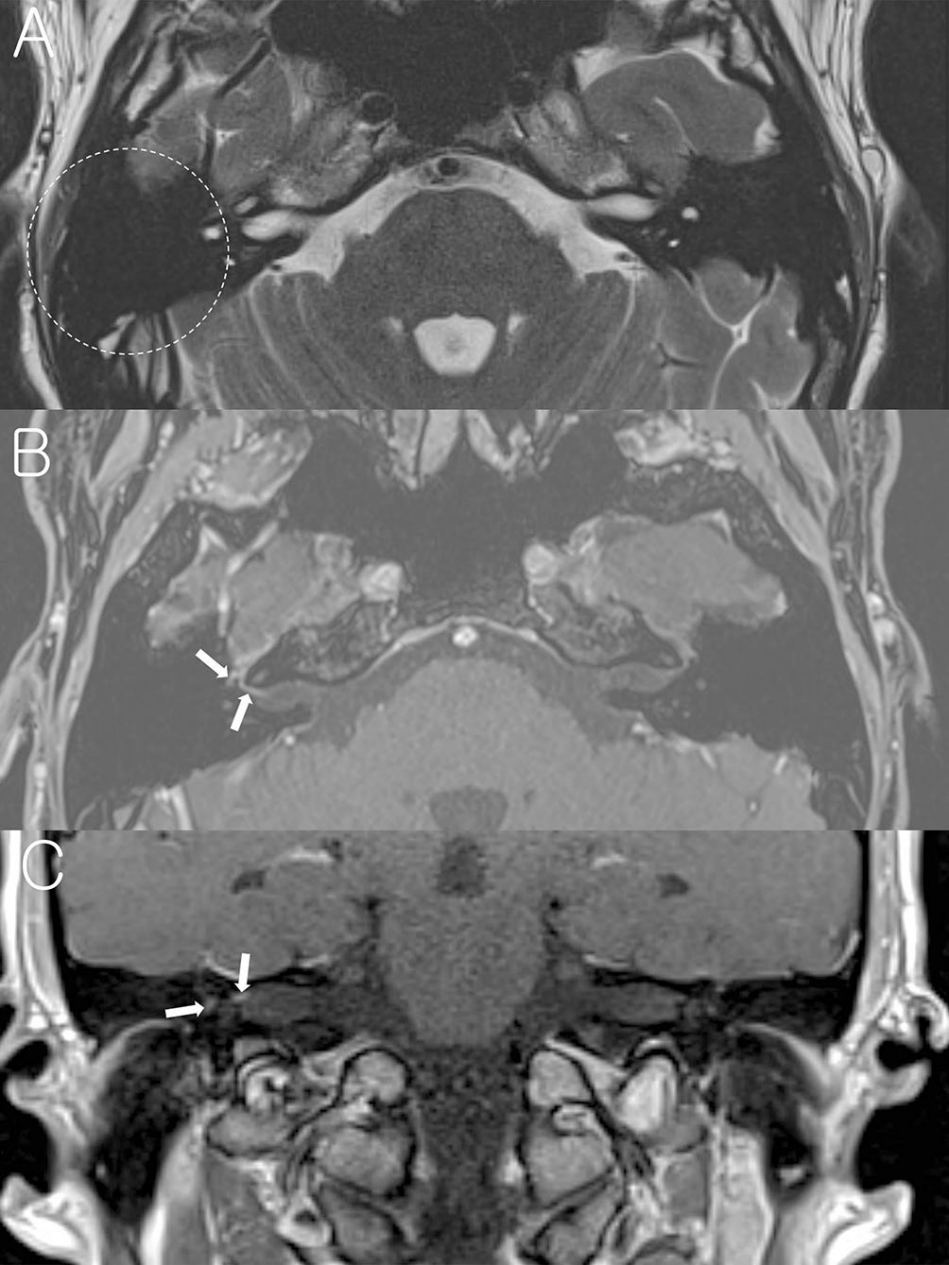
A representative case of the right Bell’s palsy with normal mastoid cavity in temporal bone MRI. (**A**) Non-contrast axial T2-weighted image shows normal mastoid cavity on the right side (dotted circle). (**B**) Post-contrast axial T1-weighted image at the Bill’s bar shows enhancement of the distal canalicular, labyrinthine, genu and proximal tympanic segment of the right facial nerve (arrows). (**C**) Post-contrast coronal T1-weighted image at the level of IAC demonstrates enhancement of the right facial nerve.

**Figure 2:**
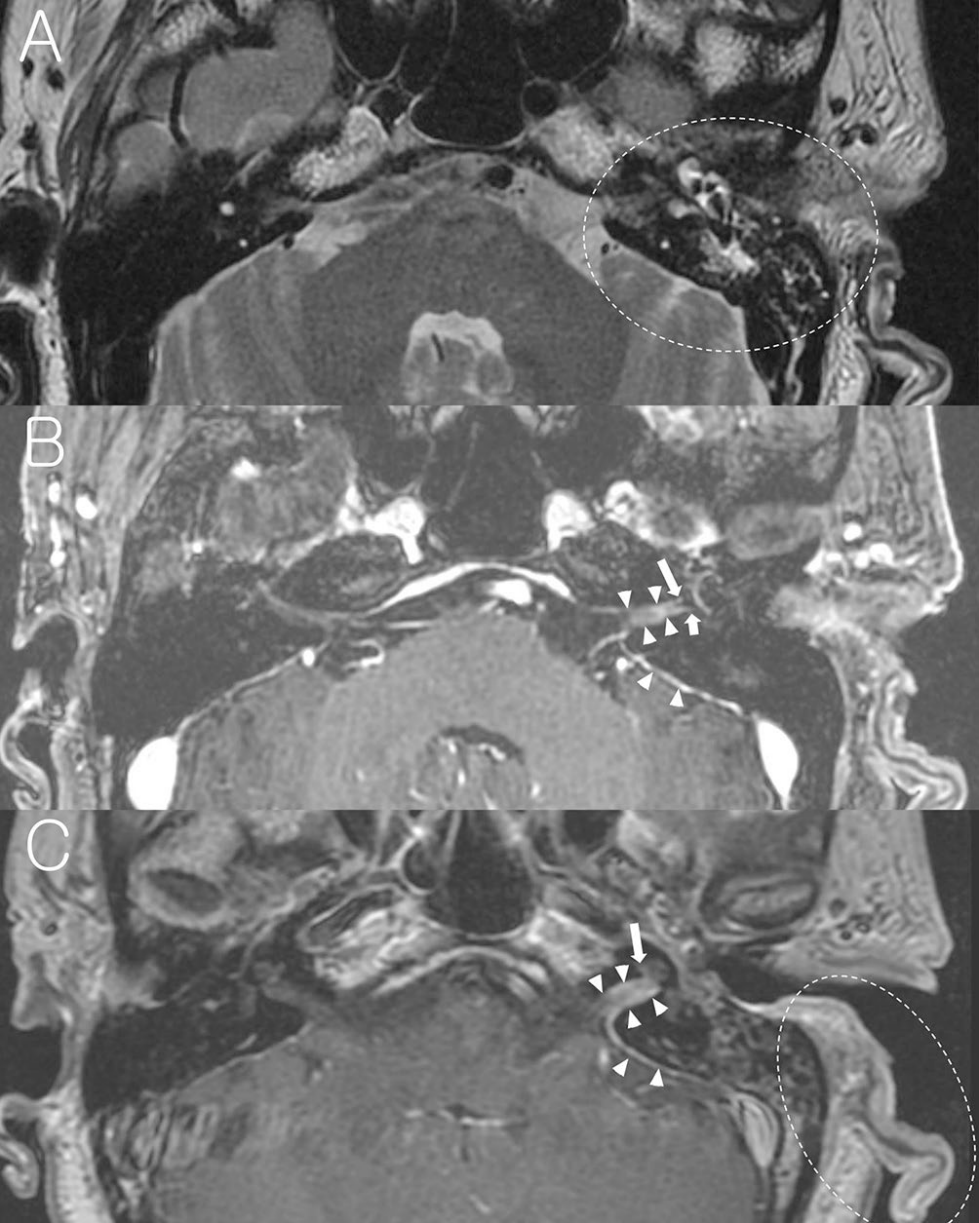
A representative case of Ramsay Hunt syndrome with mastoid effusion on the left side. (**A**) Non-contrast axial T2-weighted image at the level of IAC and mastoid air cells shows high signal intensity from the fluid in the left mastoid suggesting mastoid effusion (dotted circle). (**B**) Post-contrast axial T1-weighted image at the Bill’s bar shows enhancement of the distal canalicular and labyrinthine segment of the left facial nerve (long arrow), and superior vestibular nerve (short arrow). The adjacent dura in fundic area of the left IAC and left posterior petrous ridge are also enhanced (arrowheads). (**C**) Post-contrast axial T1-weighted image at the level of IAC demonstrates mild enhancement of basal turn of the left cochlea and diffuse dural enhancement along the whole length of the left IAC and left posterior petrous ridge (arrow heads). Left auricle is thickened and enhanced (dotted circle) as compared with right side.

**Figure 3:**
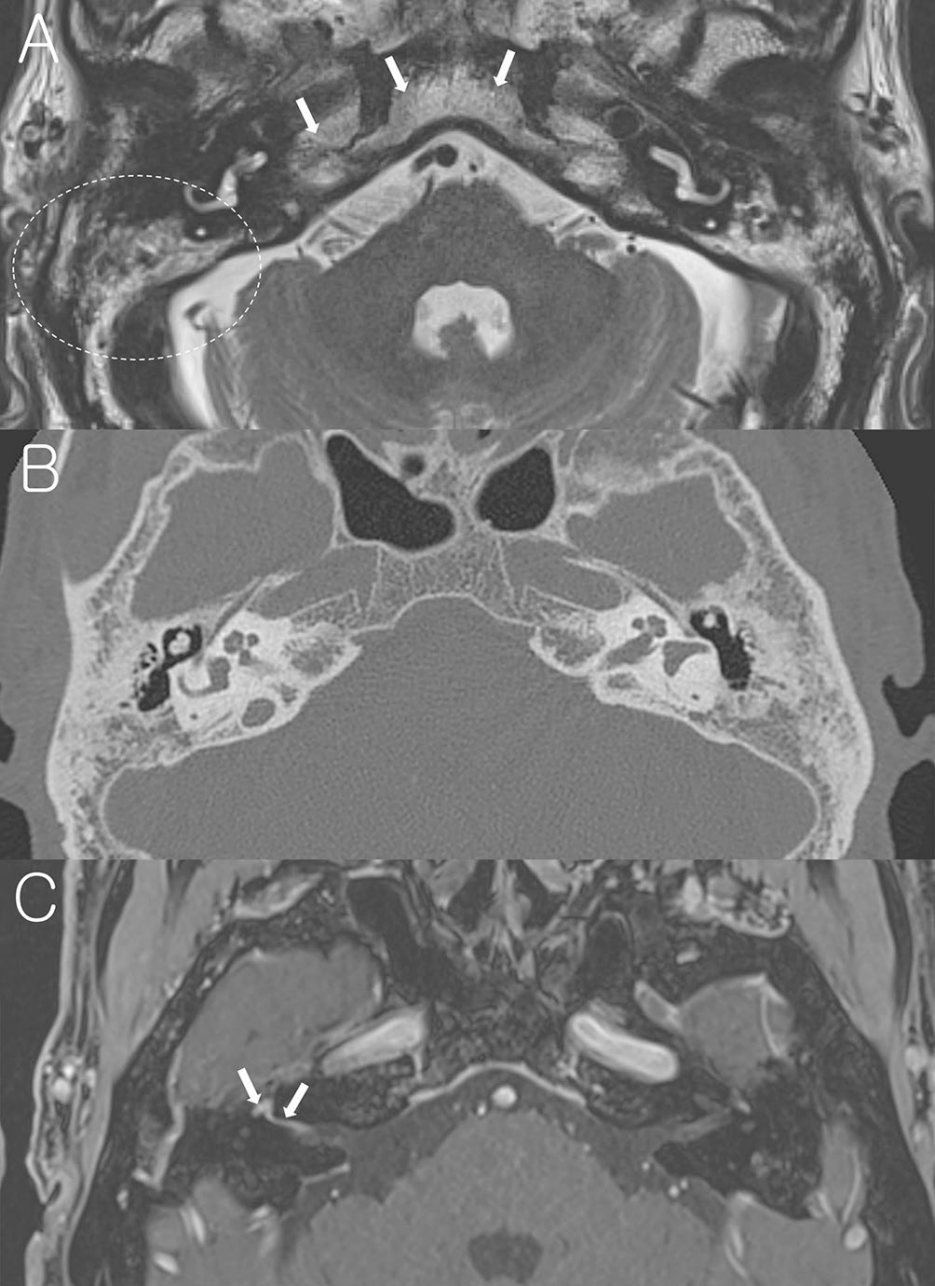
A representative case of Bell’s palsy with sclerotic mastoid. (**A**) Non-contrast axial T2-weighted image at the level of IAC shows heterogeneous high signal intensity in the mastoids (dotted circle) which have similar signal intensity from the adjacent clivus and petrous apex (arrows), suggesting bony sclerotic change. (**B**) Temporal bone CT confirms bony sclerotic change and decreased number of mastoid air cells. (**C**) Post-contrast axial T1-weighted image at the Bill’s bar shows enhancement of the distal canalicular, labyrinthine, genu and proximal tympanic segment of the right facial nerve (arrows).

While previous studies investigated the imaging findings of facial nerve, inner ear end organs, dura, and vestibulocochlear nerve in patients with acute peripheral FNP^13–20^, the present study focused on the mastoid cavity in the affected side on temporal bone MRI. Findings of mastoid cavity were classified into three groups as normal mastoid (Fig. 1), mastoid effusion (Fig. 2), and sclerotic change (Fig. 3). Figure 1 demonstrates a representative case with normal mastoid in BP patient on the right side. T2-weighted axial image shows normal appearance with no high signal intensity (Fig. 1A). Post-contrast axial and coronal T1-weighted images show enhancement of the right facial nerve at the distal canalicular, labyrinthine, geniculate ganglion, and proximal tympanic segments (Fig. 1B, C). Figure 2 demonstrates a representative case with mastoid effusion in RHS patient on the left side. Non-contrast axial T2-weighted image at the level of internal auditory canal (IAC) and mastoid air cells shows high signal intensity from the fluid in the left mastoid suggesting mastoid effusion (Fig. 2A). Post-contrast axial T1-weighted image at the Bill’s bar shows enhancement of the distal canalicular and labyrinthine segment of the left facial nerve, and superior vestibular nerve. The adjacent dura in fundic area of the left IAC and left posterior petrous ridge are also enhanced (Fig. 2B). Post-contrast axial T1-weighted image at the level of IAC demonstrates mild enhancement of basal turn of the left cochlea and diffuse dural enhancement along the whole length of the left IAC and left posterior petrous ridge. The left pinna is thickened and enhanced as compared with the right side (Fig. 2C). Figure 3 demonstrates a representative case with sclerotic mastoid in a patient with BP on the right side. Axial T2-weighted image depicts heterogeneous high signal intensity in the mastoids which have similar signal intensity from the adjacent clivus and petrous apex, suggesting bony sclerotic change (Fig. 3A). Temporal bone computed tomography (TBCT) confirms the bony sclerotic change with decreased number of mastoid air cells (Fig. 3B). Post-contrast axial T1-weighted image at the Bill’s bar shows enhancement of the distal canalicular, labyrinthine, geniculate ganglion and proximal tympanic segment of the right facial nerve (Fig. 3C).

**Figure 4:**
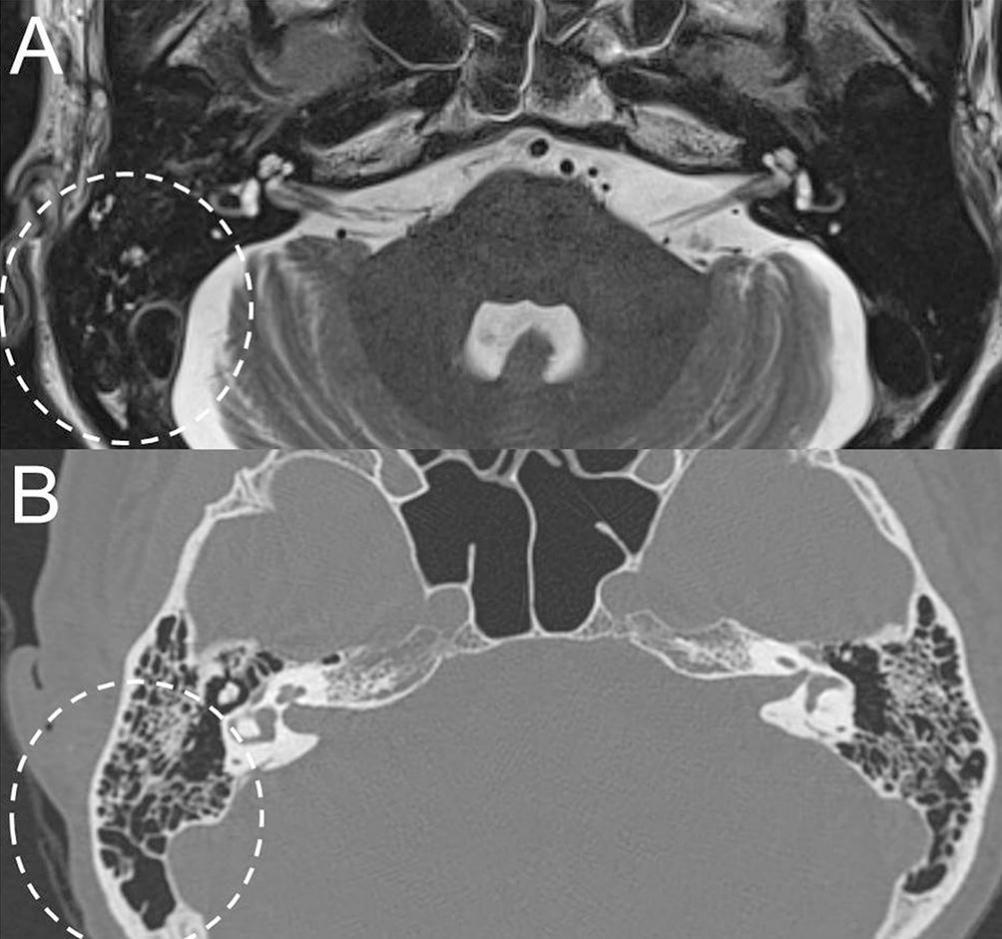
Comparison between temporal bone MRI and TBCT in a representative case of Ramsay Hunt syndrome with mastoid effusion on the right side. Non-contrast axial T2-weighted image at the level of IAC and mastoid air cells shows high signal intensity from the fluid in the right mastoid suggesting mastoid effusion (dotted circle, **A**), which was not easily identifiable in TBCT (dotted circle, **B**).

Normal mastoid cavity in temporal bone MRI was observed in 98% (128 of 131) of BP and 48% (16 of 33) of RHS, and ipsilesional mastoid effusion was observed in 0% of BP and 42% (14 of 33) of RHS (Table 2). Bony sclerotic change was observed in 2% (3 of 131) of BP and 9% (3 of 33) of RHS, and the sclerotic change was observed in both mastoid cavities in all patients. It has been suggested that the extent of mastoid pneumatization is determined genetically, rendering its variation an anatomical and etiological factor for chronic otitis media^22^, or determined by environmental factors such as pathologic influences on the middle ear and mastoid air cell system during childhood^23^. Because no evidence for chronic otitis media was suspected in any of six patients with the sclerotic mastoid cavity in temporal bone MRI and the sclerotic change was observed not only on the same side with FNP but also on the contralateral side (Fig. 3A, B) in all six patients, those patients were excluded from the statistical analysis, given that sclerotic bony change in the mastoid is not associated with the acute onset of FNP. Proportion of mastoid effusion was significantly higher in RHS (47%, 14 of 30) than BP (0%, 0 of 128) (*P* < 0.001, Pearson’s chi-squared test). We compared the proportion of mastoid effusion according to the time between symptom onset of FNP and temporal bone MRI in patients with RHS. Among 14 RHS patients with mastoid effusion, temporal bone MRI was performed within 3 days after onset of acute FNP in 11 patients and after 3 days of onset of acute FNP in 3 patients. Among 16 RHS patients without mastoid effusion, temporal bone MRI was performed within 3 days after onset of acute FNP in 12 patients and after 3 days of onset of acute FNP in 4 patients. The proportion of the patients, in whom temporal bone MRI was conducted within 3 days after onset of acute FNP, was 78.6% (11 of 14) in RHS patients with mastoid effusion and 75% (12 of 16) in those without mastoid effusion, which was not significantly different (*P* = 0.817, Pearson’s chi-squared test). Then, we compared the severity of FNP between RHS patients with normal mastoid and those with mastoid effusion. The worst HB grade during an acute stage was 3.4 ± 0.8 in RHS with normal mastoid (*n* = 16) and 3.8 ± 1.2 (*n* = 14) in those with mastoid effusion, which was not significantly different (*P* = 0.201, Mann – Whitney *U* test; Table 3). Air–bone gap was not revealed on a pure tone audiometry in any of 14 RHS patients with mastoid effusion. Otoendoscopic examination revealed normal tympanic membrane in 7 patients (of 14 RHS patients with mastoid effusion on temporal bone MRI), and injected tympanic membrane without recognizable middle ear effusion in 7 patients (of 14 RHS patients with mastoid effusion on temporal bone MRI). TBCT or brain CT, which was conducted in 6 patients of 14 RHS patients with mastoid effusion on temporal bone MRI, demonstrated too small amount of mastoid effusion to be easily detected (Fig. 4).

**Table 2:** Temporal bone MRI findings in the mastoid area.

	BP (*n* = 131)	RHS (*n* = 33)
Normal	128 (98%)	16 (48%)
Effusion	0 (0%)	14 (42%)
Sclerotic change	3 (2%)	3 (9%)

MRI, magnetic resonance imaging; BP, Bell’s palsy; RHS, Ramsay Hunt syndrome.

**Table 3:** Initial House-Brackmann grade according to magnetic resonance imaging (MRI) findings of the mastoid area in Bell’s palsy (BP) and Ramsay Hunt syndrome (RHS).

	MRI finding in mastoid area		
	Normal	Mastoid effusion	
**House-Brackmann grade**			
BP (*n* = 128)	3.2 ± 0.8 (*n* = 128)	NA (*n* = 0)	
RHS (*n* = 30)	3.4 ± 0.8 (*n* = 16)	3.8 ± 1.2 (*n* = 14)	*P* = 0.201

NA, not applicable.

**Figure 5:**
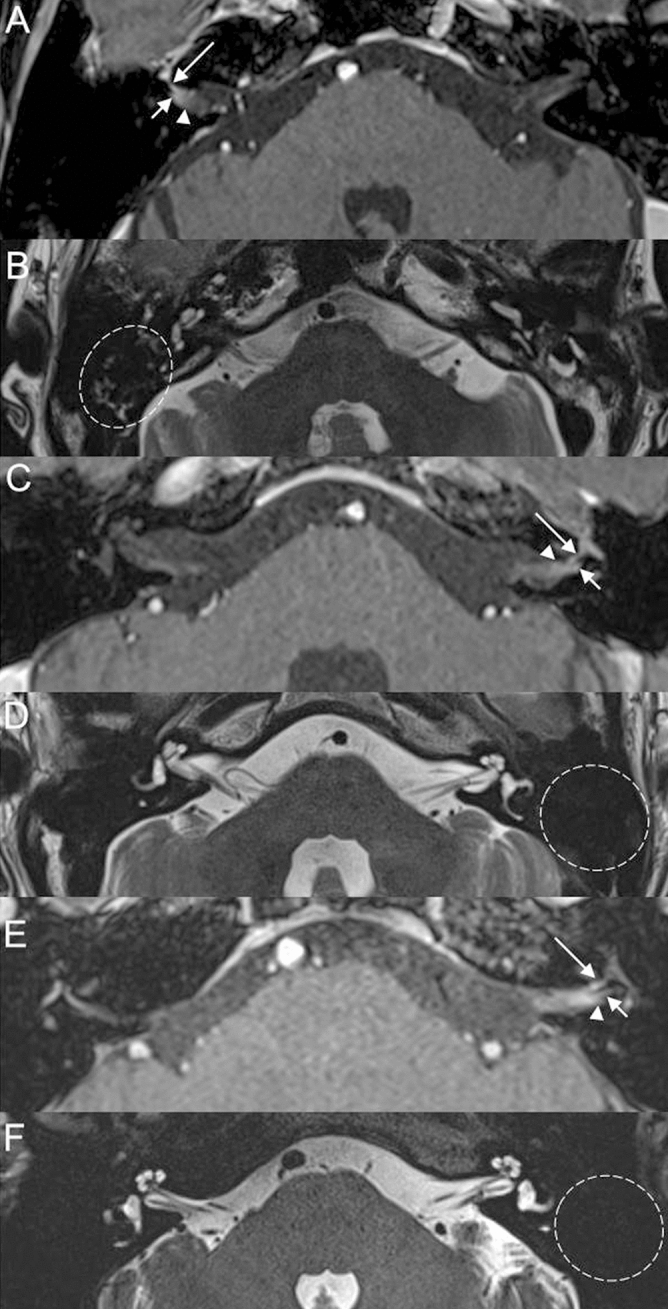
Temporal bone MRI of the patient with zoster sine herpete in the right side (**A**, **B**). (**A**) Post-contrast 3D T1 weighted image at the level of the internal auditory canal demonstrates enhancement of the labyrinthine segment of the right facial nerve (long arrow), right vestibular nerve (short arrow), and dura (arrowhead). (**B**) Non-contrast axial T2-weighted image shows high signal intensity from the fluid in the right mastoid suggesting mastoid effusion (dotted circle). Temporal bone MRI of the patient, who developed vesicular skin eruption in the left ear without acute facial nerve palsy (**C**, **D**). (**C**) Post-contrast 3D T1 weighted image at the level of the internal auditory canal demonstrates enhancement of the labyrinthine segment of the left facial nerve (long arrow), left vestibular nerve (short arrow), and dura (arrowhead). (**D**) Non-contrast axial T2-weighted image shows normal mastoid cavity on the left side (dotted circle). Temporal bone MRI of another patient, who developed vesicular skin eruption in the left ear without acute facial nerve palsy (**E**, **F**). (**E**) Post-contrast 3D T1 weighted image at the level of the internal auditory canal demonstrates enhancement of the labyrinthine segment of the left facial nerve (long arrow), left vestibular nerve (short arrow), and dura (arrowhead). (**D**) Non-contrast axial T2-weighted image shows normal mastoid cavity on the left side (dotted circle).

In the present study, only RHS patients with typical symptoms of acute FNP with vesicular skin eruption were included (see Subjects and Methods). We then evaluated temporal bone MRI findings of another three atypical RHS patients who did not show vesicular eruption or acute FNP (Fig. 5). Temporal bone MRI of a 64-year old female patient, who was diagnosed as zoster sine herpete in the right side, is shown in Fig. 5A, B. Postcontrast 3D T1-weighted MRI at the level of the IAC demonstrated the enhancement of the labyrinthine segment of the right facial nerve, right vestibular nerve and IAC dura (Fig. 5A). Non-contrast axial T2-weighted image at the level of mastoid air cells showed high signal intensity suggesting mastoid effusion on the affected side (Fig. 5B). Temporal bone MRI of a 40-year-old male patient, who developed vesicular skin eruption in the left ear and acute vertigo without acute FNP, is shown in Fig. 5C, D. Postcontrast 3D T1-weighted MRI showed the enhancement of the labyrinthine segment of the left facial nerve, left vestibular nerve and IAC dura (Fig. 5C). Non-contrast axial T2-weighted image showed normal mastoid cavity in the affected side (Fig. 5D). Temporal bone MRI of a 28-year-old male patient, who developed vesicular skin eruption in the left ear and acute vertigo and hearing loss without acute FNP, is shown in Fig. 5E, F. Postcontrast 3D T1-weighted MRI showed the enhancement of the labyrinthine segment of the left facial nerve, left vestibular nerve, and IAC dura (Fig. 5E). Non-contrast axial T2-weighted image at the level of mastoid air cells showed normal mastoid cavity in the affected side (Fig. 5F).

## Discussion

The present study demonstrates that the significantly higher incidence of mastoid effusion was observed in RHS than BP on temporal bone MRI. Previous studies have emphasized that early differential diagnosis between RHS and BP is important due to their different prognoses and treatment modalities^5,7–10,24,25^. However, differentiating RHS from BP based on clinical presentation at an early stage is sometimes difficult in RHS cases with atypical clinical manifestations or zoster sine herpete ^11,12,15,26^.

Temporal bone MRI has been utilized for the early differential diagnosis between BP and RHS ^13–20^. While the enhancement is generally localized within the facial nerve in BP, the enhancement, as shown in the present study (Fig. 2), has been observed in not only the facial nerve but also the vestibulocochlear nerve on temporal bone MRI in RHS^12–15,20,27–32^. Enhancement of the inner ear organs has been frequently observed in RHS^12,13,15,19,20,27,29,30,33^, and dural enhancement along the IAC, as shown in the present study (Fig. 2), has also been reported^15,20,27,28,30,33^. In RHS with multiple cranial neuropathy, enhancement of involved cranial nerves in the brainstem has been observed in temporal bone MRI^15,34^. Soft tissue swelling and enhancement of the involved auricle has been reported in temporal bone MRI of RHS patients^15,30^. Kuya et al. reported that 3D MRI sequences are useful for the differential diagnosis between RHS and BP, and RHS, compared to BP, shows more thickening of the facial nerve in the fundus of IAC on 3D-contructive interference on steady state sequence (3D-CISS) image^20^. Sugiura et al. reported that high signal intensity of the inner ear organs is observed on non-contrast 3D-fluid-attenuated inversion recovery (3D-FLAIR) images in RHS^16^.

In addition to the above-mentioned characteristic findings of temporal bone MRI in RHS, the presence of mastoid effusion in temporal bone MRI may provide an additional information in differentiating RHS from BP, because, though not all of RHS patients showed mastoid effusion (14 of 33 RHS patients), mastoid effusion was not observed in any of the patients with BP. In addition, among three RHS patients with atypical clinical manifestations, one patient with zoster sine herpete showed mastoid effusion on the affected side. It has been reported that incidental mastoid effusion on MRI, in which isolated fluid in the mastoid bone appears with absent corroborating clinical features, may have little clinical significance^35–38^. However, mastoid effusion, which was observed in 14 patients with RHS in the present study, may be associated with RHS, because the mastoid effusion was observed on the same side with RHS in all of 14 patients. The mechanism underlying mastoid effusion in RHS is not clear. We speculate that impairment of autonomic nervous function, which is thought to play a role in the regulation of local blood flow and glandular secretion in middle ear and mastoid mucosa, is responsible for the production of mastoid effusion in patients with RHS^39^.

While we performed this study with 3.0 T MRI only, it has been reported that facial nerve enhancement can also be detected on 1.5 T MRI with high spatial resolution in pathologic conditions such as BP, RHS and tumorous lesions including lymphoma and leukemia^40^. However, because higher spatial resolution and more sensitivity to the contrast materials can be achieved by using the 3.0 T MRI, we can detect facial nerve enhancement in pathologic conditions more rapidly and sensitively. Moreover, it is more convenient to differentiate enhancement between facial, vestibulocochlear nerve and dura by using the 3.0 T MRI. However, considering that mild to moderate enhancement of the intrameatal (15%) and labyrinthine (5%) segments of the normal facial nerve may be observed on 3.0 T MRI^41^, attention should be paid in the interpretation of the presence of facial nerve enhancement by comparing the pattern and degree of the bilateral facial nerve enhancement on MRI.

## Conclusions

In conclusion, this study highlighted significantly higher incidence of ipsilesional mastoid effusion in RHS than BP, and suggested that the presence of mastoid effusion may provide additional information for differential diagnosis between RHS and BP. In addition to enhancement of vestibulocochlear nerve and/or dura along the IAC and/or inner ear organs in temporal bone MRI, identification of mastoid effusion may be a valuable MRI finding for early differential diagnosis.

## Data Availability

The raw/processed data required to reproduce these findings cannot be shared at this time as the data also forms part of an ongoing study.
